# Efficacy of a newly developed guidewire for selective biliary access

**DOI:** 10.1038/s41598-023-34846-w

**Published:** 2023-05-11

**Authors:** Do Hyun Park, Joung-Ho Han, Tae Hoon Lee, Jae Kook Yang, Ji Sung Lee, Yong Hun Lee, Mamoru Takenaka, Sang-Heum Park

**Affiliations:** 1 Department of Internal Medicine, Asan Medical Center, University of Ulsan College of Medicine, Seoul, South Korea; 2 Department of Internal Medicine, Chungbuk National University Hospital, Chungbuk National University College of Medicine, Cheongju, South Korea; 3 Department of Internal Medicine, Soonchunhyang University Cheonan Hospital, Soonchunhyang University College of Medicine, 31, Sooncheonhyang 6-gil, Dongnam-gu, Cheonan, Chungcheongnam-do 31151 South Korea; 4 Clinical Research Center, Asan Institute for Life Sciences, Asan Medical Center, University of Ulsan College of Medicine, Seoul, South Korea; 5 Department of Clinical Epidemiology and Biostatistics, Asan Medical Center, University of Ulsan College of Medicine, Seoul, South Korea; 6 Research and Development, Sungwon Medical Co., Ltd., Cheongju, South Korea; 7 Department of Gastroenterology and Hepatology, Faculty of Medicine, Kindai University, Osaka, Japan

**Keywords:** Gastroenterology, Medical research

## Abstract

A clinical efficacy study of 0.025-inch guidewires (GWs) according to mechanical property analysis has not been reported yet. This study was designed to evaluate the clinical efficacy of a newly developed 0.025-inch GW for biliary access according to the basic mechanical property. Commercially available 0.025-inch GWs were in vitro tested based on parameters of mechanical property. Patients with naïve papilla requiring diagnostic or therapeutic ERCP were randomly assigned to an experimental 0.025-inch newly developed GW or a control 0.025-inch GW group. Technical success rate of wire-guided cannulation (WGC), difficult biliary cannulation (DBC), and adverse event rates were measured in this multicenter randomized trial. The technical success rate of primary WGC was 79.1% (151 of 191) in the experimental group and 70.8% (131 of 185) in the control group (95% two-sided confidence interval: 8.25%; *p* < 0.001; for a noninferiority margin of 15%). The technical success rate including cross-over to each other was also non-inferior. However, the chi-square test showed a statistical difference (81.7% *vs.* 68.1%; *p* = 0.002). Median biliary cannulation time was shorter in the experimental group (53 s *vs.* 77 s; *p* = 0.047). The rate of DBC was more frequent in the control group (34.6% *vs.* 50.3% *p* = 0.002). Multivariate analysis revealed that control group was one of contributing factors for DBC. Overall rate of post-ERCP pancreatitis was not different (4.7% *vs.* 8.6%; *p* = 0.125). WGC using a newly developed GW with superior physical performance GW in a bench test showed similar clinical efficacy and the rate of DBC was significantly lower in experimental GW.

## Introduction

Guidewire (GW) for endoscopic retrograde cholangiopancreatography (ERCP) was developed to maintain access to common bile duct (CBD) during therapeutic ERCP procedures such as pneumatic balloon dilatation, stent placement, device exchange, or guidance for stone removal^1^. Currently GW is also widely used to facilitate primary selective biliary or pancreatic cannulation and pass tight ductal strictures^2, 3^. Especially, as a procedure related factor, the use of GW during biliary cannulation might have a technical advantage and ability to reduce the frequency or severity of post-ERCP pancreatitis. Wire-assisted cannulation technique can increase the primary cannulation success rate and reduce the risk or severity of post-ERCP pancreatitis compared with the standard contrast-injection method^2–9^. European Society of Gastrointestinal Endoscopy (ESGE) recommends the guidewire-assisted technique for primary biliary cannulation since it can reduce the risk of post-ERCP pancreatitis^2^.

However, technical success of selective biliary cannulation is also affected by other numerous factors^2, 5^. Successful biliary cannulation is influenced by operator level of experience and patient related factors such as the morphology or anatomy of the major papilla, periampullary diverticulum, or altered anatomy. Since patient-related factors cannot be changed, if the operator's experience level is similar, the use of a GW might have technical advantages. Therefore, numerous types of GWs have been developed and the standard 0.035-inch GW has been used for decades.

Recently, the use of 0.025-inch GWs has gradually increased. A 0.025-inch GW provides thinner diameter with similar core diameter compared with a 0.035-inch GW. It provides similar pushing ability to a 0.035-inch GW. However, it allows for an easier device exchange. In addition, it is easy to rotate a GW within a papillotome or bile duct. A thinner GW with a hydrophilic coating tip also has the ability to facilitate through severe strictures^3, 5, 10^.

However, the selection of an optimal GW might be difficult because numerous GWs from each manufacturer have different features. Clinical selection of GWs usually depends on the preference of the operator, availability, and/or the type of procedure^3, 10^. Studies that compare each basic property of GWs and clinical comparative studies are limited because of heterogenicities, especially for 0.025-inch GWs.

In this bench test and subsequent clinical trial, we analyzed basic mechanical properties of currently available 0.025-inch GWs. We also tried to evaluate a new 0.025-inch endoscopic GW with enhanced properties compared with conventional 0.025-inch GWs. This GW has a thin hydrophilic coating and high torque ability with a 0.025-inch stiff core to facilitate transpapillary biliary access. The purpose of this trial was to determine the rate of primary technical success and clinical outcomes without additional procedures for selective biliary access between a newly developed GW and a conventional GW based on the best mechanical performance among 0.025-inch GWs in a previous bench test.

## Methods

### Basic property tests of guidewires

**Figure 1:**
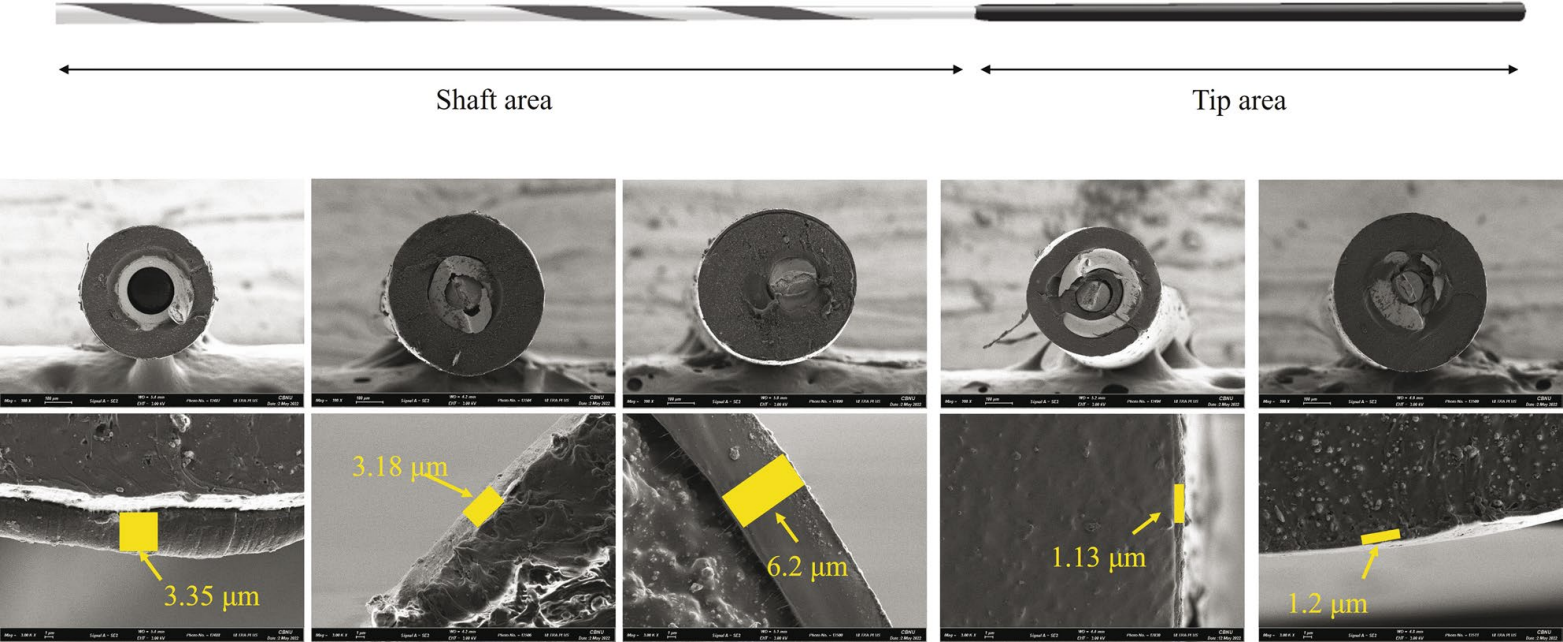
Typical characteristics of guidewires. (**A**, upper) Structure of a TRUwire. The guidewire is structurally divided into a shaft area and a tip area. The hydrophilic coating is mainly applied for the tip area. (**B**, lower) Scanning electron microscopy (ULTRA PLUS; zeiss group., Land Baden-Württemberg, Germany) cross-section images of guidewires (× 100, upper line) and hydrophilic coating thickness (× 3,000, lower line): TRUwire, VisiGlide 2, Jagwire Revolusion, Acrobat II, MICHISUJI (Left to right). Spiral coiled spring structure was noted on all guidewires except Jagwire.

The GW for ERCP is structurally divided into two parts: a rigid shaft to support or guide various accessories and a hydrophilic tip area to track and enter the desired path. The recently used ERCP GWs use a monofilament or spiral coil spring for the tip portion of the Nikel Titanum alloy core, which is a shape memory alloy with a hydrophilic coating on the surface to reduce friction (Fig. 1).

Basic characteristics of commercially available 0.025 GWs tested prior to clinical study are described in Table 1, including the following: TRUwire (0.025-inch diameter, 450-cm length, angled and straight tip; Sungwon Med. Corp., Korea); VisiGlide 2™ (0.025-inch diameter, 450-cm length, angled and straight tip; Olympus Co., Tokyo, Japan); Jagwire™ Revolution (0.025-inch diameter, 450-cm length, angled and straight tip; Boston Scientific, Marlborough, MA, USA); Acrobat II™ (0.025-inch diameter, 450-cm length, curved and straight tip; Cook Endoscopy, USA); and MICHISUJI (0.025-inch diameter, 450-cm length, curved and straight tip; KANEKA Medical, Osaka, Japan). Basic size, configuration, and materials of tested GWs were mostly similar, including core material, sheath material, tip coating, and spiral coiled spring of tip except Jagwire.

**Table 1:** Basic characteristics of 0.025-inch guidewires tested prior to clinical study.

Commercial name	Length (cm)	Tip length (mm)	Width of the tip (mm)	Core material	Sheath material	Tip core material	Tip coating	Spiral coiled spring	Shape of tip
TRUwire	450	72	0.532	Nitinol	PTFE	Platinum	Hydrophilic polyurethane	Yes	Straight or angled
VisiGlide 2	450	71	0.595	Nitinol	Fluorine coating polyethylene	Confidential	Hydrophilic PTFE	Yes	Straight or angled
Jagwire Revolution	450	50	0.607	Nitinol	PTFE	Tungsten	Hydrophilic polyurethane	No	Straight or angled
Acrobat II	450	51	0.501	Nitinol	PTFE	Platinum	Hydrophilic polyurethane	Yes	Straight or angled
MICHISUJI	450	150	0.565	Nitinol	PTFE	Gold	Hydrophilic polyurethane	Yes	Straight or angled

PTFE, polytetrafluoroethylene.

**Figure 2:**
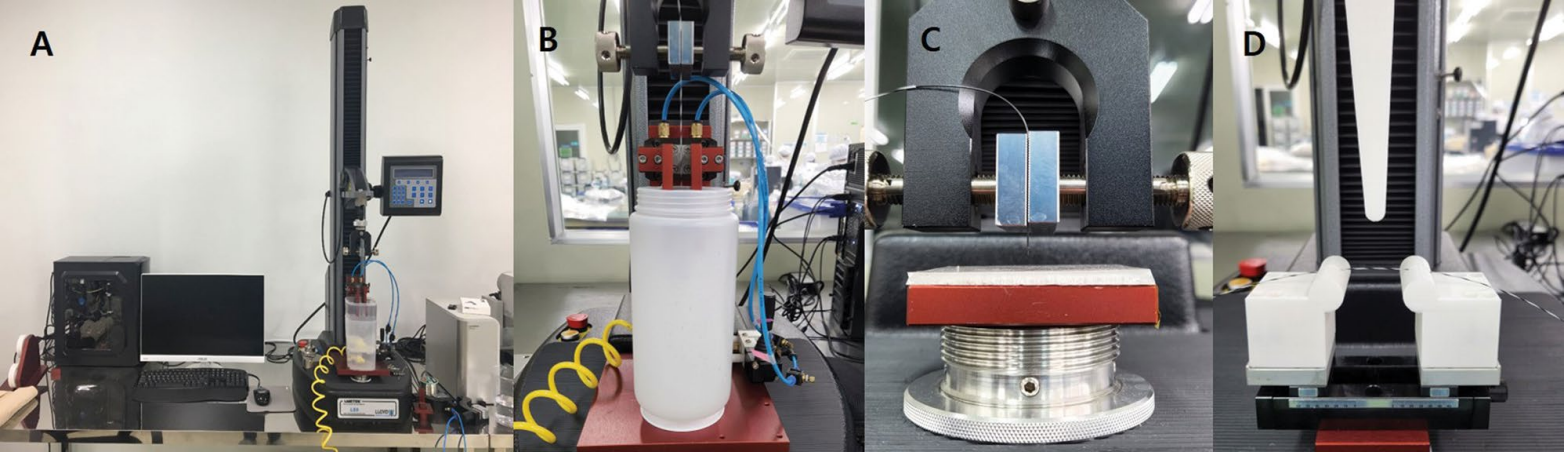
Basic property tests for guidewires (Left to right; **A** to **D**). (**A**) A universal testing machine (LS1; LLOYD instrument Ltd., West Sussex, England) for the tip load, tip friction, bending test. (**B**) Tip Friction test jig: Dipping a silicon pad and wire in a bath, compressing the silicone pad with the wire at a pressure of 3 Bar, and then stretching them by 20 mm at a speed of 20 mm/min. (**C**) Tip load test jig: Compressing a wire by 2 mm at a speed of 5 mm/min over the silicone pad after fixing the part 10 mm away from the tip. (**D**) Bending Force test jig: Compressing a wire to 20 mm at a speed of 20 mm/min after extending the jig by 60 mm and fixing the wire.

Therefore, we measured tip friction, tip load, and bending force using a universal test machine (LS1; LLOYD Instrument Ltd., West Sussex, England) (Fig. 2a). First, the tip friction was measured by dipping a silicon pad and wire in a bath, compressing the silicone pad with the wire at a pressure of 3 Bar, and then stretching them by 20 mm at a speed of 20 mm/min (Fig. 2b). Second, the tip load was measured by compressing a wire by 2 mm at a speed of 5 mm/min over the silicone pad after fixing the part 10 mm away from the tip of the wire with a jig (Fig. 2c). Finally, the bending force was measured by compressing a wire to 20 mm at a speed of 20 mm/min after extending the jig by 60 mm and fixing the wire (Fig. 2d). Results of all tests were obtained by averaging measured values of three attempts for each of five different GWs.

### Results of basic property tests

**Figure 3:**
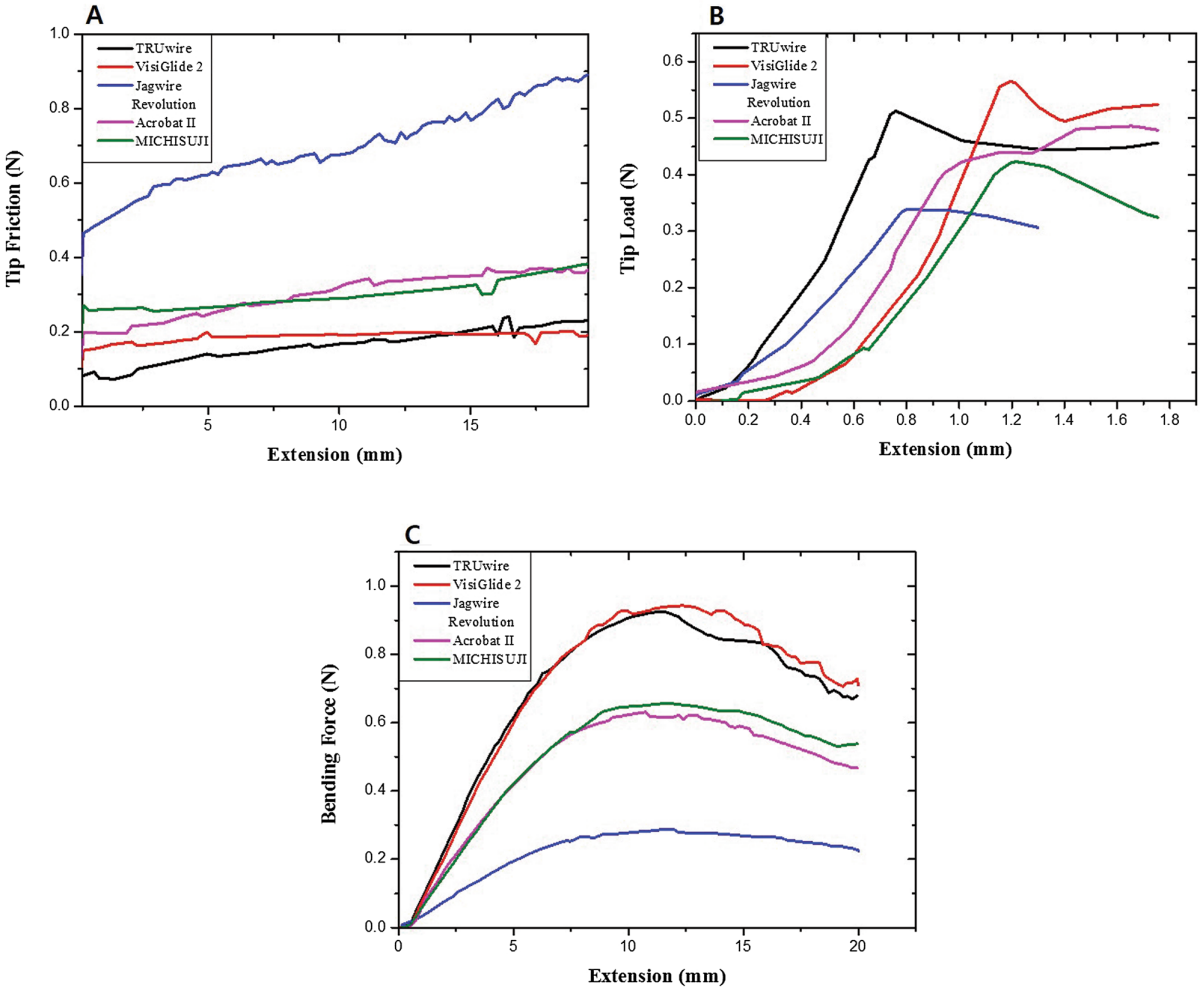
Results of basic property tests (Left to right; **A** to **C**). (**A**) Tip friction test: TRUwire showed relatively lower N value. (**B**) Tip load test: Maximal loading force was different according to the extension. Mean force was higher in VisiGlide 2. (**C**) Bending force test: Change in the bending force according to the displacement of the bending point. TRUwire and VisiGlide 2 showed similar force values.

TRUwire had the lowest tip friction, while Jagwire Revolution had the highest tip friction (Fig. 3a). VisiGlide 2 had the highest tip load and bending force, while Acrobat II had the lowest tip load and bending force (Fig. 3b,c, and Table 2). Mean values of tip friction, load, and bending force are described in Table 2. It was also confirmed that values of tip friction, tip load, and bending force were different depending on the internal structure and coating thickness on scanning electron microscope (SEM) images (Fig. 1 and Table 2). The bending force of the tip of GWs could not be measured because of the uncheckable and extremely low bending force by this machine.

**Table 2:** Mean values of estimated mechanical tests.

Guidewires	Average tip friction (N)	Maximum tip load (N)	Maximum bending force (N)	Average coating thickness (μm)
TRUwire	0.16 ± 0.046	0.51 ± 0.013	0.92 ± 0.003	3.35 ± 0.21
VisiGlide 2	0.18 ± 0.025	0.52 ± 0.035	0.95 ± 0.023	3.18 ± 0.03
Jagwire Revolution	0.76 ± 0.174	0.50 ± 0.014	0.62 ± 0.005	6.2 ± 1.41
Acrobat II	0.28 ± 0.063	0.34 ± 0.019	0.28 ± 0.005	1.13 ± 0.12
MICHISUJI	0.28 ± 0.007	0.38 ± 0.043	0.66 ± 0.02	1.2 ± 0.41

Data are presented as mean ± standard deviation.

VisiGlide 2 and TRUwire showed similar properties in tip friction, tip load, and bending force. Even if the outer diameter of the tip and shaft of the 0.025- inch GWs were similar, the lower the tip friction and the higher the tip load, the more useful it might be to enter the desired biliary lesion and find a way after entering. Also, the higher the bending force, the more useful it could be to maintain the path for insertion or exchange of biliary stents or balloons. Based on these experimental bench tests, we assumed that the newly developed 0.025-inch GW would show similar performances with a presented higher performance GW, VisiGlide 2 in selective biliary cannulation during ERCP.

### Clinical study protocol

**Figure 4:**
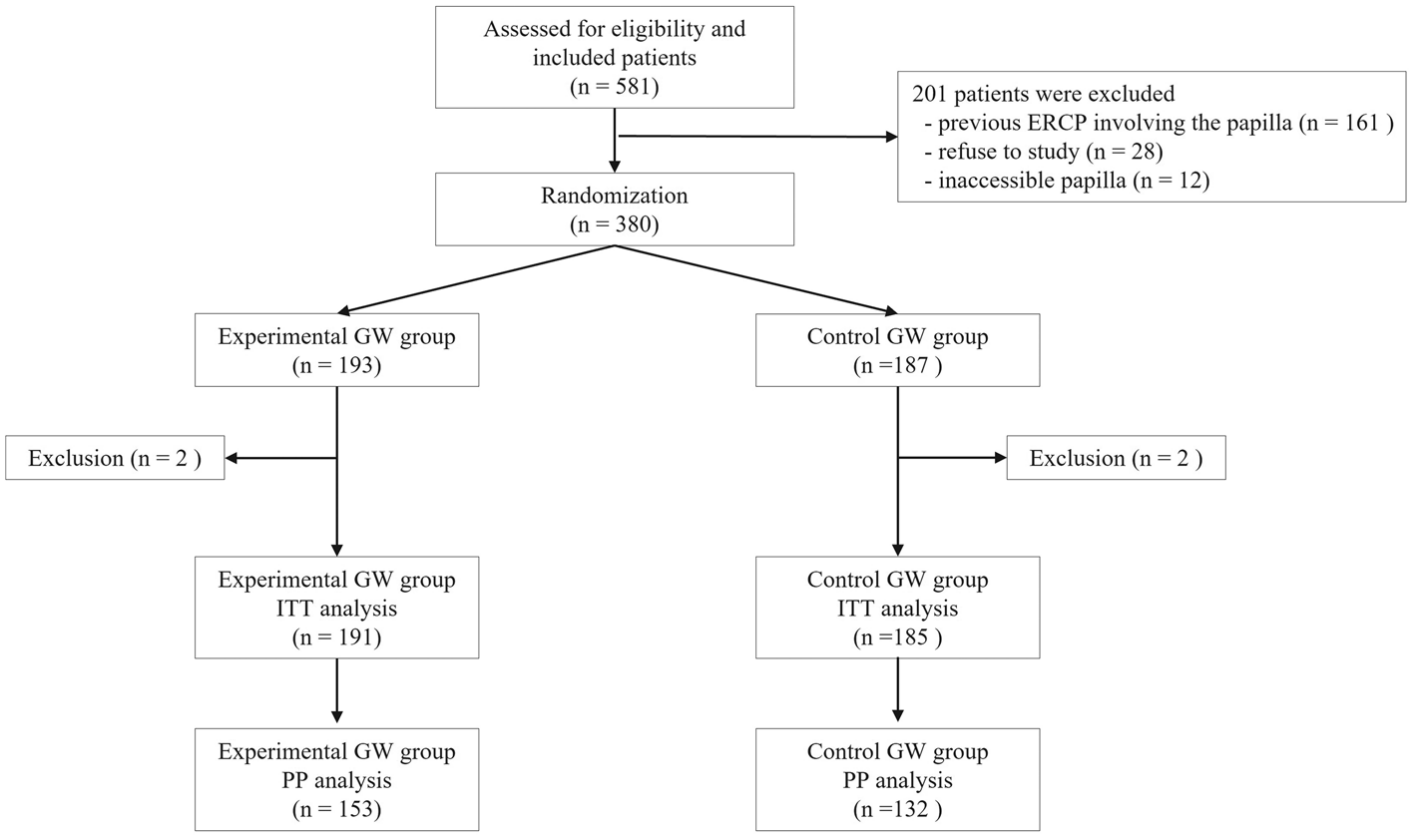
Flow of presented study.

This study was designed as a prospective, multicenter, randomized, non-inferiority trial. It was conducted in three tertiary referral university hospitals (Soonchunhyang University, Asan Medical Center, and Chungbuk University) in South Korea. Enrolled patients were randomized into an experimental or a control group without risk stratification at a 1:1 ratio from March 15, 2021 to January 27, 2022 (Fig. 4). We obtained sequentially numbered, opaque, sealed envelopes with computer-generated random numbers. Patients were blinded to procedures that they had undergone. Endoscopists were also blinded to treatment arms before successful WGC because typical patterning of the shaft on each guidewire was not seen during WGC using a preloaded guidewire in a papillotome or a cannula by an assistant nurse for whom allocated treatment arms. All authors had access to the study data and reviewed and approved the final manuscript. Our study was performed in accordance with the ethical guidelines of the 1975 Declaration of Helsinki. Written informed consent was obtained from each participant after full explanation. Institutional Review Boards of Soonchunhyang University Cheonan Hospital, Asan Medical Center, and Chungbuk National University Hospital approved this study. This trial was then registered in cris.nih.go.kr (number KCT0005696; registered date: 23/12/2020).

### Study subjects

Patients with naïve papilla who were candidates for diagnostic or therapeutic biliary ERCP were invited to participate in this study. All patients underwent endoscopic ultrasonography (EUS), abdominal computed tomography, or magnetic resonance image (or magnetic resonance cholangiopancreatography) before ERCP. Exclusion criteria were as follows: age less than 18 years, presenting acute pancreatitis before ERCP, endoscopic papillectomy, EUS-guided intervention accessing bile duct, inaccessible papilla due to altered anatomy, previous history of pancreatitis, and allergy to contrast.

### Endoscopic procedures

After an overnight fast, all patients underwent ERCP in the prone position with a standard duodenoscope (TJF 260, Olympus Optical, Tokyo, Japan) after sedation with intravenous midazolam (0.05 mg/kg) and/or propofol (0.5 mg/kg). Prophylactic antibiotics and analgesics were permitted. Prophylactic rectal NSAID was not used in this study because of unavailability of this drug in Korea. All ERCP procedures were performed by experienced four endoscopists (> 3000 career ERCPs with a workload of at least 350 ERCPs annually) without involvement of trainees. The GW was controlled by experienced subspecialized endoscopy nursing staffs with at least two years of training.

Wire-guided cannulation (WGC) of the bile duct was undertaken using a long-wire technique. For a WGC, a hydrophilic-tipped GW (TRUwire, Sungwon Med. Corp., Korea or VigiGlide 2, Olympus, Japan) with a diameter of 0.025 inch was preloaded into a conventional cannula (Glo-Tip® ERCP Catheter, GT-1-T, Cook Medical, Bloomington, IN, USA) or pull-type sphincterotome (CleverCut3V sphincterotome, Olympus, Japan; Tri-Tome pc®, Cook Medical, Bloomington, IN, USA). The cannula or papillotome was oriented from the 11 to 12 o’clock position on the papilla and bowed to align it correctly with the axis of the bile duct. After a minimal insertion (2–3 mm) of the pull-type papillotome or cannula in the ampulla, the GW was carefully advanced through the CBD under fluoroscopy until it was seen to enter the bile duct (Touch technique)^11^. If the pancreatic duct (PD) was entered, the GW was simply withdrawn. Attempts to redirect it toward the CBD were made. After biliary cannulation was achieved through guidewire insertion, contrast injection was allowed.

In cases of difficult biliary cannulation (DBC) according to the ESGE guideline^2^, persistent GW cannulation up to 10 min, double-guidewire cannulation (DGC) technique, precut fistulotomy with a needle-knife (Microtome, Boston Scientific, Microvasive, Marlboro, MA, USA), or CBD access following placement of PD stent was used at the discretion of the endoscopist. Endoscopic sphincterotomies for therapeutic purpose were performed in both groups after access to the CBD. A PD stent for prophylactic prevention of pancreatitis was not used routinely in this study.

### Objective and definitions

The primary endpoint was primary intended technical success of WGC for successful biliary access. Secondary endpoints were overall technical success of biliary cannulation, rate of DBC, cannulation time, adverse event rates, and procedure outcomes. Mechanical properties of each 0.025-inch GWs before clinical trials were also analyzed. DBC was defined by the presence of one or more of the followings: more than five contacts with the papilla whilst attempting to cannulate; more than five minutes spent attempting to cannulate following visualization of the papilla; and more than one unintended pancreatic duct cannulation or opacification according the ESGE suggestion^2^. Papillary shape was defined according to previous results^12^.

Serum amylase level was measured before ERCP and at 2 h and 12–24 h after ERCP. Hyperamylasemia after ERCP was defined as an elevation of the serum amylase level above the upper normal limit (160 IU/L). Definition and grade of post-ERCP pancreatitis was as follows: new or worsened abdominal pain with elevation of serum amylase at least 3 times above the upper normal limits for 24 h after a procedure that requires at least 2–3 days (mild), 4–10 days (moderate), and more than 10 days (severe) of hospitalization or if any of the following occurred: hemorrhagic pancreatitis, pancreatic necrosis, pseudocyst, or the need for percutaneous drainage or surgery. Adverse events and severity of pancreatitis were described according to the lexicon for endoscopic adverse events^13^.

### Statistical analysis

A total of 342 patients (1:1 randomization) were planned for evaluation as the intention-to-treat (ITT) population. Assuming a 10% drop-out rate, the final sample size was calculated to be 380 patients (190 per group). With these patient numbers and under the assumption that technical success rate would be 90% in control group and 75% experimental group, the study had approximately 80% power to show non-inferiority of the experimental group compared to the control group at a one-sided significance level of 0.025, with a lower confidence bound for the difference in technical success rate greater than − 15%. The non-inferiority margin was based on results of a pooled analysis and after discussion with contributing physicians who stated that this noninferiority margin of 15% would be clinically relevant^11, 14^. Because there was no reported comparing study of 0.025-inch GWs, as a first clinical trial, we planned a non-inferiority study.

Primary and secondary endpoints were assessed in the ITT population, which included patients who were randomized. Per-protocol analysis was additionally performed for patients who had no additional rescue techniques in DBC. Values are presented as mean ± standard deviation (SD), median (interquartile range [IQR]), range for continuous variables, or as the number (%) of subjects for categorical variables. For primary endpoint, the primary hypothesis of non-inferiority of the experimental group relative to control group was tested first. If non-inferiority was established, a superiority test was done without multiplicity adjustments. Secondary and safety endpoint analyses were conducted using Pearson Chi-square test, Fisher's exact test, or Wilcoxon rank sum test as appropriate as indicated. To determine meaningful variables for DBC, odds ratios (ORs) and their 95% confidence intervals (CIs) were calculated using multivariable logistic regression analysis. All reported *p* values are two-sided. All statistical analyses were done using SAS version 9.4 (SAS Institute Inc., Cary, NC, USA). *P*-values of less than 0.05 were considered to indicate statistical significance.

## Results

### Baseline characteristics

Baseline characteristics of the two groups are summarized in Table 3. Regarding the number of subjects from each participating center, there were 135 subjects from Soonchunhyang University Hospital, 127 from Asan Medical Center, and 118 from Chungbuk University Hospital, showing no significant difference between the two groups. Common indications for ERCP in both groups were CBD stones, cholangiocarcinoma, and pancreatic cancer. Comorbid diseases, frequency of periampullary diverticulum, and papillary shape were not significantly different between the two groups.

**Table 3:** Baseline characteristics of study subjects.

N (%)	Experimental (TRUwire) (n = 191)	Control (VisiGlide 2) (n = 185)	*P*-value
Age (mean ± SD)	65.3 ± 14.0	66.7 ± 12.8	0.297
Sex			0.627
Male/Female	108 (56.5)/83 (43.5)	100 (54.1)/85 (45.9)	
Diagnosis			0.316
CBD stone	110 (57.6)	110 (59.5)	
Cholangiocarcinoma	24 (12.6)	30 (16.2)	
Pancreas cancer	15 (7.9)	17 (9.2)	
Metastatic biliary obstruction	12 (6.3)	8 (4.3)	
Gallbladder cancer	8 (4.2)	3 (1.6)	
Hepatocellular carcinoma	4 (2.1)	4 (2.2)	
Ampullary cancer	3 (1.6)	3 (1.6)	
Benign biliary obstruction	6 (3.1)	0	
Post-operative injury	1 (0.5)	1 (0.5)	
Sphincter of Oddi dysfunction	0	1 (0.5)	
Others	8 (4.2)	8 (4.3)	
Previous cholecystectomy	25 (13.1)	22 (11.9)	0.725
Comorbid diseases, n (%)			
Cardiovascular	88 (46.1)	79 (42.7)	0.510
Cerebral	22 (11.5)	15 (8.1)	0.267
Diabetes	48 (25.1)	42 (22.7)	0.581
Chronic kidney disease	2 (1.0)	9 (4.9)	0.028
Liver cirrhosis	10 (5.2)	6 (3.2)	0.338
Others	15 (7.9)	18 (9.7)	0.520
No comorbid diseases	63 (33.0)	75 (40.5)	0.128
Biliary obstruction level			0.604
Perihilar	15 (7.9)	24 (13)	
I/ II/ IIIA/ IIIB/ IV	2/ 4/ 2/ 4/ 3	4/ 2/ 7/ 3/ 8	
Proximal CBD	8 (4.2)	7 (3.8)	
Mid-CBD	8 (4.2)	8 (4.3)	
Distal-CBD	52 (27.2)	49 (26.5)	
Absent	108 (56.5)	97 (52.4)	
Periampullary diverticulum			0.432
Absent	142 (74.3)	129 (69.7)	
*Type I/II/III	15 (7.9)/ 19 (9.9)/ 15 (7.9)	24 (13.0)/ 19 (10.3)/ 13 (7.0)	
CBD diameter, mm (mean ± SD)	12.1 ± 4.8	11.7 ± 4.6	0.433
Papillary shape			0.290
Non-prominent	113 (59.2)	105 (56.8)	
Prominent	51 (26.7)	54 (29.2)	
Bulging hooknose	13 (6.8)	19 (10.3)	
Distorted	14 (7.3)	7 (3.8)	
Initial lab			
Total bilirubin, median (IQR)	1.5 (0.6–4.5)	2.1 (0.8–6.1)	0.062
Hb, mean ± SD	12.7 ± 2.1	12.3 ± 1.7	0.061
ALT, median (IQR)	84.0 (29.0–217.0)	105.0 (41.0–264.0)	0.110
Amylase, median (IQR)	46.0 (30.0–72.0)	51.0 (32.0–84.0)	0.155
Lipase, median (IQR)	38.5 (27.0–69.0)	46.0 (31.0–72.0)	0.077

*P*-value by Pearson chi-square test, Fisher’s exact test, Student's t-test, or Wilcoxon rank sum test as appropriate.

CBD, common bile duct; SD, standard deviation; IQR, interquartile range.

*Type I (extradiverticulum); II (juxtadiverticulum); III (intradiverticulum).

### Main outcomes measurements

Primary technical success rate of WGC was 79.1% (151 of 191) in the experimental group and 70.8% (131 of 185) in the control group (95% two-sided confidence interval, 8.25%; *p* < 0.001; for a noninferiority margin of 15%). Technical success rate including cross-over to each other was also non-inferior. However, Chi-square test showed a statistically significant difference (81.7% *vs.* 68.1%; *p* = 0.002). In per-protocol analysis, technical success rate of experimental group was also non-inferior (*p* < 0.001) (Table 4).

**Table 4:** Main procedure outcomes (Intention-To-Treat and Per-Protocol analysis).

N (%)	Experimental (n = 191)		Control (n = 185)		Difference in proportion (%)(two-sided 95% CI)	*P*-value†	*P*-value‡
Technical success of primary WGC	151 (79.1)		131 (70.8)		8.25 (− 0.48–16.98)	< .001	0.064
Technical success including cross-over	156 (81.7)		126 (68.1)		13.57% (4.90–22.24)	< .001	0.002
Technical success of primary WGC (PP)	151/153 (98.7)		131/132 (99.2)		− 0.55% (− 2.88–1.78)	< .001	1.000
Technical success including cross-over (PP)	152 (99.3)		130 (98.5)		0.86% (− 1.58–3.31)	< .001	0.597
Overcoming methods in difficult cannulation							
Precut fistulotomy	22 (11.5)		32 (17.3)				0.110
Double-guidewire cannulation	1 (0.5)		3 (1.6)				0.365
Biliary access after PD	16 (8.4)		23 (12.4)				0.197
Overall cannulation success	186 (97.4)		181 (97.8)		− 0.46 (− 3.54–2.63)	< .0001	1.000
Cannulation method							0.908
Conventional cannula	65 (34)		64 (34.6)				
Papillotome	126 (66)		121 (65.4)				
Frequency of papillary contacts, median (IQR) [range]	2 (1–4) [1, 20]		2 (1–4) [1, 16]				0.240
Cannulation time°, median (IQR) [range]	53 (22–180) [3, 1506]		77 (30–240) [1, 2848]				0.047
Cannulation time°, median (IQR) [range] (PP)	42 (20–115) [3, 400]		45 (23–115) [1, 833]				0.446
Total procedure time*, median (IQR) [range]	545 (360–927) [120, 3610]		562 (400–900) [180, 3514]				0.420
GW looping during cannulation							0.244
None	125 (65.4)		131 (70.8)				
in CBD	66 (34.6)		53 (28.6)				
in PD	0		1 (0.5)				
GW type	Straight	Angled	Straight	Angled			0.868
	78 (40.8)	113 (59.2)	74 (40)	110 (60)			
Technical success of primary WGC	65 (83.3)	86 (76.1)	56 (75.7)	75 (67.6)			0.100
DBC, difficult biliary cannulation	24 (30.8)	42 (37.2)	36 (48.6)	57 (51.4)			0.015

WGC, wire-guided cannulation; CBD, common bile duct; PD, pancreatic duct; GW, guidewire; PP, per protocol analysis; IQR, interquartile range.

From ampulla of Vater access to CBD access.

*From ampulla of Vater access to completion of procedure.

^†^ *P*-value for non-inferiority test using z-statistics with non-inferiority margin of 15%.

^‡^ *P*-value by Pearson chi-square test, Fisher's exact test, or Wilcoxon rank sum test as appropriate.

Patients who failed primary WGC due to DBC underwent precut, DGC, or CBD access after placement of PD stent. The ratio of each technique was not significantly different between the two groups. Overall cannulation success rates including these rescue techniques were 97.4% and 97.8%, respectively (*p* = 1.00). Total procedure time was not different. However, the median cannulation time was shorter in the experimental group (53 s *vs.* 77 s; *p* = 0.047). According to the type of each guidewire (straight versus angled), primary technical success tended to be higher in the straight type without a statistical difference (*p* = 0.100). However, the rate of DBC was higher in the control group regardless of straight or angled type (*p* = 0.015) (Table 4).

### Analysis for difficult biliary cannulation

**Figure 5:**
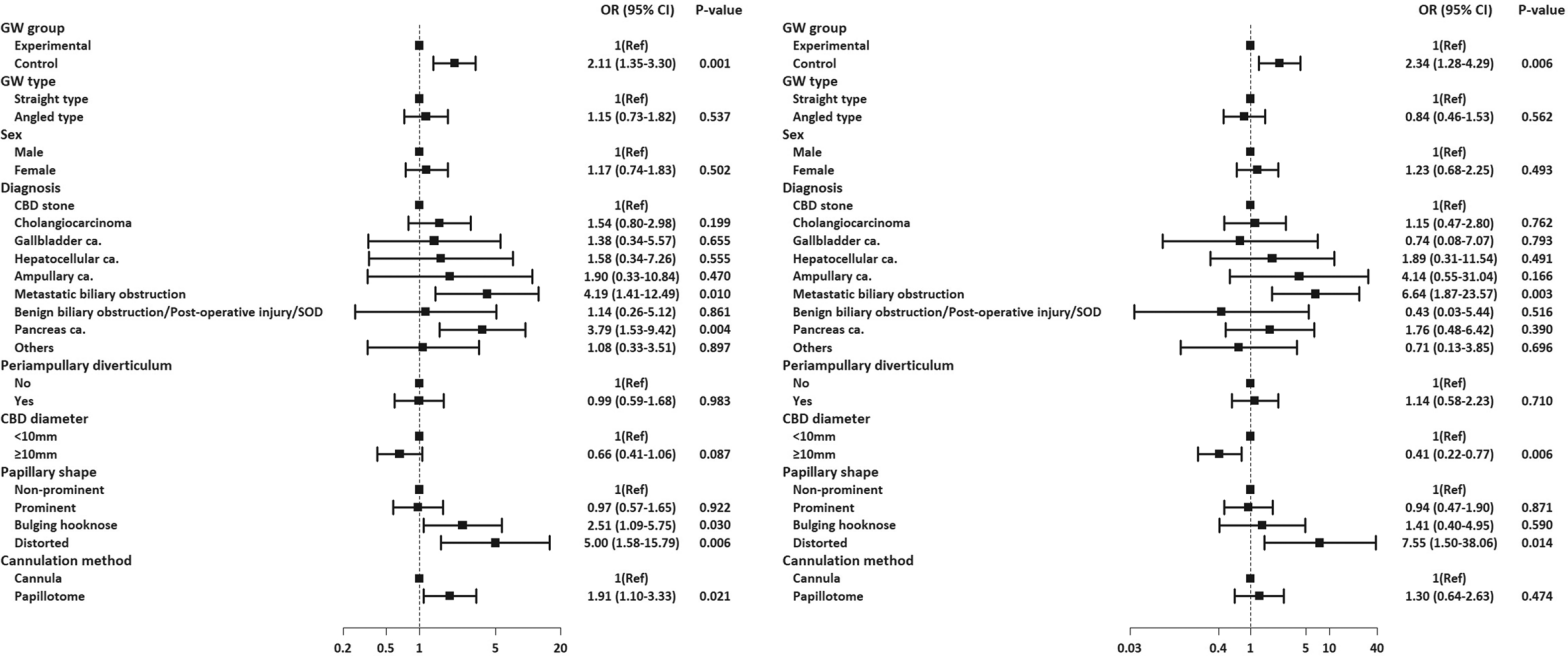
Multivariate logistic regression in Intention-To-Treat (left) and Per-Protocol (right) analysis for factors affecting difficult biliary cannulation.

In the analysis of DBC, the rate of DBC was more frequent in the control group (34.6% *vs.* 50.3% *p* = 0.002). PD cannulation or opacification was more common in the control group. Subsequently, persisting WGC, DGC, precut, or CBD access after PD stent placement was done. There was no difference between the two groups. Cross-over the GW into another group was tried more commonly in the control group (Table 5). Multivariate logistic regression in ITT for factors of DBC revealed that control group, bulging or distorted type of papilla, cannulation method using a papillotome, and disease category for metastatic biliary obstruction and pancreas cancer were meaningful factors for DBC (Fig. 5).

**Table 5:** Analysis of difficult biliary cannulation and procedure outcomes.

N (%)	Experimental (n = 191)	Control (n = 185)	*P*-value
DBC, total	66 (34.6)	93 (50.3)	0.002
Papillary contacts ≥ 5	34 (17.8)	41 (22.2)	0.290
Cannulation time ≥ 5 min	36 (18.8)	41 (22.2)	0.426
PD cannulation or opacification ≥ 1	40 (20.9)	70 (37.8)	0.0003
Next step in DBC			
Ongoing WGC	27 (14.1)	37 (20)	0.130
GW exchange†	5 (2.6)	7 (3.8)	0.072
Straight → Angle type in same group	4 (80)	1 (14.3)	
Exchange with another group	1 (20)	6 (85.7)	
DGC with pancreatic sphincterotomy	1 (0.5)	3 (1.6)	0.365
Precut fistulotomy	22 (11.5)	32 (17.3)	0.110
CBD access after PD stent placement	16 (8.4)	23 (12.4)	0.197
Biliary stricture	81 (42.4)	79 (42.7)	0.954
Technical success of biliary stricture passage	80 (98.8)*	79 (100)	1.000
No. of attempts, median (IQR) [range]	1 (1—1) [0, 14]	1 (1—2) [0, 15]	0.321
Use GW only	69 (85.2)	65 (82.3)	0.618
GW with papillotome	11 (13.6)	13 (16.7)	0.586
Looping during passage of stricture	46 (54.8)	37 (45.7)	0.243
Asymptomatic hyperamylasemia	23 (12)	12 (6.5)	0.063
Post-ERCP Pancreatitis	9 (4.7)	16 (8.6)	0.125
Mild/ moderate/severe	9 (4.7)/0/0	14 (7.6)/2 (1.1)/0	
GW-related pancreatitis	1 (0.5)	2 (1.1)	0.618

*Failure case: percutaneous transhepatic drainage.

†GW exchange during ongoing WGC.

DBC, difficult biliary cannulation; WGC, wire-guided cannulation; GW, guidewire; CBD, common bile duct; DGC, double-guidewire cannulation; PD, pancreatic duct; IQR, interquartile range.

### Other outcomes and adverse events

The rate of biliary stricture due to malignant or benign obstruction was not different. The technical success of stricture passage using a GW was not different between the two groups either (Table 5). Overall endoscopic procedures after biliary access were not different between the two groups. There were no serious adverse events or mortality in either group. Asymptomatic hyperamylasemia was 12% in the experimental group and 6.5% in control group (*p* = 0.063). Overall rate of post-ERCP pancreatitis was 4.7% (9 of 191) in the experimental group and 8.6% (16 of 185) in the control group (*p* = 0.125). Only two patients in the control group had a moderate level of pancreatitis. GW-related pancreatitis was one and two patients in each group, respectively (Table 5).

## Discussion

Nowadays, GW is widely used for facilitating selective biliary access and reducing the rate or severity of post-ERCP pancreatitis. However, the clinical efficacy study of GWs during biliary cannulation has some limitations because GWs can vary in diameter, body stiffness, tip shape, and flexibility according to the manufactures. Selection of GWs is usually decided by the preference of physicians and the availability of manufactures. Although such GWs have been devised and commercialized, clinical studies on 0.025-inch GWs based on mechanical properties of each GW are insufficient.

Therefore, we first planned this clinical trial according to the basic property analysis of GWs, although there were limitations to conducting clinical studies with the purpose of obtaining an objective understanding of new product characteristics. The strength of the present study was that it was a randomized trial following mechanical property analysis of available 0.025-inch GWs. According to the best results of mechanical property analysis including a newly developed GW in experimental group, we compared it with a high performance GW, VisiGlide 2 previously studied in numerous reports^15–17^, which had higher performance characteristics in our technical properties.

In basic mechanical property analysis of 0.025-inch GWs (Table 1), VisiGlide 2 and TRUwire showed similar results. However, VisiGlide 2 GW showed relatively higher performance in tip load test and bending force test, while TRUwire revealed relatively lower friction force (Table 2 and Fig. 3). Based on these characteristics, we planned a comparative study with a non-inferiority design.

Our clinical trial revealed that the primary WGC success rate of experimental GW was non-inferior and the overall technical success was not different from that of the control GW, VisiGlide 2. However, when we analyzed the primary outcome including the cross-over, primary technical success was higher in the experimental GW group (Chi-square test). This difference was associated with the rate of DBC, which was statistically lower in the experimental GW group. DBC according to the criteria can be developed in various situations such as patients’ factor, physician’s experience, or devices. Baseline characteristics were not different. The level of physicians’ experience was also similar. Multivariate logistic regression showed that conventional GW was also a meaningful negative factor affecting the DBC. Although results of mechanical property test for experimental and conventional GW were similar, the friction force was relatively lower in the experimental GW group. Lower friction force in the tip of GW might influence DBC because complex mucosal features of intrapapillary ducts including columnar-shaped mucosal reduplications and pocket-like mucosal folds might be the main reasons for DBC during mechanical contact of tip of GW to duodenal major papilla^18^. According to the tip core material and coating, technical difficulty might be a factor influencing DBC. However, there is a limitation to prove this difference with concrete data.

Regarding adverse events, another primary concern of this study was the rate of post-ERCP pancreatitis. It was not different between the two groups because of a relatively lower rate of pancreatitis. However, slightly higher tendency was shown in the control group, although it was not statistically significant. DBC rate might result in this tendency.

WGC is now popular as the initial biliary cannulation method instead of traditional contrast injection method. ESGE guideline also suggests WGC as a primary cannulation method. WGC can facilitate technical success rate of selective biliary cannulation. It may reduce the severity or frequency of post-ERCP pancreatitis^4–6, 19^. Among numerous GWs, the 0.035-inch GW used to be the preferred GW because of its stiffness. However, there has been an increase in the use of 0.025-inch GWs since they have similar core shaft stiffness of 0.035-inch GW with thinner hydrophilic coating compared with 0.035-inch GW, which might decrease the friction rate and become easy to handle^10^. One study has also reported that 0.025-inch GW (VisiGlide 2) might facilitate selective biliary cannulation compared to conventional 0.035-inch GW (Jagwire; Boston Scientific, Natick, MA, USA) in terms of reducing cannulation time and papilla attempts^15^. The success rate of primary biliary cannulation showed a tendency to be higher in the 0.025-inch group than in the 0.035-inch GW group. Another study has compared the efficacy between 0.025 and 0.035-inch GWs and found no difference in technical success for biliary cannulation or adverse events^20, 21^. These results on overall successful WGC rates of 0.025-inch GW were similar to our results for conventional 0.025-inch WG. However, these previous reported studies compared different sized GWs. More studies on GWs considering various factors including technical success, adverse events and other clinical outcomes are needed. When we analyze the differences clinically, we also need to know more basic mechanical properties of GWs.

Therefore, in our study, we tried to compare mechanically superior GWs with each other. Previous studies of GWs usually showed technical outcomes and adverse events without clearly showing the evidence of mechanical property difference. Besides stiff core material of 0.025-inch GWs that was similar to that of 0.035-inch GWs (Fig. 1), more detailed characteristics of GWs such as friction, tip load, and bending forces were also analyzed (Figs. 2 and 3). Lower tip friction, higher tip load, and higher bending force might be important factors for facilitating selective biliary access in or negotiation of stricture. According to this difference, clinical results might be different. It can be used as a further study for the development of GWs.

Limitations of presented clinical study are as follows. First, because of a non-inferiority trial, a relatively small number of patients were enrolled. We set a margin of noninferiority for a biliary cannulation success rate of 15%, which might be a large difference. However, the estimated large number of patients to show the efficacy of these two GWs was difficult because there were no comparative studies between 0.025-inch GWs. Further superiority trials or large comparative studies are warranted. Second, we compared only one control GW because multiple comparison of GWs was limited. To overcome this limitation, we analyzed technical properties of available 0.025-inch GWs for the comparison before clinical trial. However, mechanical reports cannot follow clinical efficacy exactly. Third, although this study was performed by experienced endoscopists and nursing staff, preference of second procedures and timing might be different. Finally, available 0.025-inch GWs might be different according to countries.

In conclusion, WGC using a newly developed GW showed similar efficacy and adverse events with a higher performance than existing GW. However, the rate of DBC was relatively lower than the control GW. We found the mechanical property of a GW may affect the DBC. Therefore, ERCP physicians could recognize the mechanical property and limitations of each GWs in WGC for the reduction of DBC and prevention of possible post-ERCP pancreatitis. Also, based on tests of mechanical properties in 0.025- inch GW, we could develop more ideal GW with lower friction load, higher tip load, and higher bending force for facilitating selective cannulation or stricture negotiation.

## Data Availability

The datasets used and/or analysed during the current study available from the corresponding author on reasonable request.

## Abbreviations

ERCP: Endoscopic retrograde cholangiopancreatography
CBD: Common bile duct
PD: Pancreatic duct
GW: Guidewire
WGC: Wire-guided cannulation
DGC: Double guidewire cannulation
DBC: Difficult biliary cannulation
